# Respiratory complex I and embryo development

**DOI:** 10.1093/jxb/erw051

**Published:** 2016-02-19

**Authors:** Oren Ostersetzer-Biran

**Affiliations:** Department of Plant and Environmental Sciences, The Alexander Silberman Institute of Life Sciences, The Hebrew University of Jerusalem, Jerusalem, Israel9190401

**Keywords:** Carbonic anhydrase, CO2, embryogenesis, gamma carbonic anhydrase, membrane potential, reactive oxygen species, respiration, respiratory complex I

**Complex I (CI) is a large membranous mitochondrial enzyme that serves as the major entry point for electrons from NADH into the respiratory chain. The CI enzyme is considered to be conserved between different organisms, but plant CI includes an extra ‘egg-shaped’ module – the γ-carbonic anhydrase domain – and a paper by Córdoba *et al.* in this issue of *Journal of Experimental Botany* (pages 1589–1603) indicates that its functions are required during Arabidopsis embryogenesis.**

The respiratory machinery of the mitochondrion is usually made up of four major protein complexes, CI to IV, embedded within the inner-mitochondrial membrane. Electron transfer through CI (NADH:ubiquinone oxidoreductase), CIII and CIV mediates the pumping of protons (H^+^) across the inner membrane, forming the chemical potential (ΔpH), which is utilized by ATP-synthases to generate ATP.

CI is present in many prokaryotes, including α-Proteobacteria, the proposed progenitors of mitochondria, and is thus considered to have arisen early in evolution. The bacterial and mammalian enzymes show an L-shaped structure composed of two major fragments, including an integral membrane-domain and a soluble arm (Baradaran *et al.*, 2013; Shimada *et al.*, 2014). The plant CI consists of ≥50 different subunits, encoded by both nuclear and mitochondrial loci, forming a ≥1.0 MDa structure that has several distinguishing features (Fig. 1) (Meyer, 2012; Braun *et al.*, 2014). These include the presence of additional Nad subunits that are encoded in the mtDNA. The *nad* pre-RNAs in plant mitochondria undergo extensive maturation processes, including numerous RNA-editing events and the splicing of many group II-type introns that are removed posttranscriptionally from the coding region they interrupt (Brown *et al.*, 2014).

**Fig. 1.**
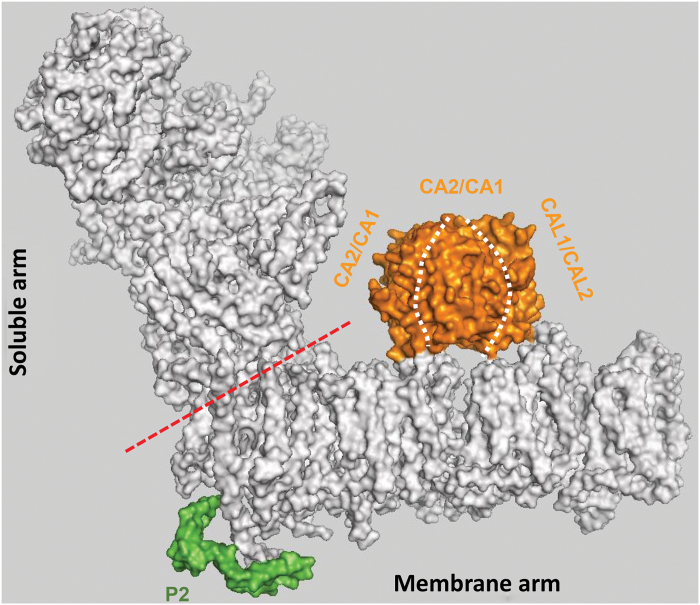
A hypothetical 3D-structural model of Arabidopsis CI. The proton-pumping CI enzyme is a large bi-partite membranous protein assembly, which has a central role in the production of cellular energy in bacteria and mitochondria. CI has a characteristic L-shaped structure with the hydrophobic arm embedded in the membrane and the hydrophilic peripheral arm facing into the cytoplasm (bacteria) or the mitochondrial matrix. The bacterial enzyme is relatively simple, containing 14 different subunits, while the mitochondrial enzymes contain extra subunits. Sequence data for the different plant CI subunits is according to Meyer (2012). Homology models were constructed individually and fitted according to the electron density map of the bovine CI enzyme (Shimada *et al.*, 2014). The approximate positions of the CA-related subunits were fitted based on the low-resolution negative stained map of Arabidopsis CI (Sunderhaus *et al.*, 2006). The solvent accessible surface for the Arabidopsis CI enzyme was visualized in Pymol (v1.3). Different subunits that are shared between the plant CI and its related bacterial and mitochondrial enzymes are shown in grey; extra subcomplex I domains of the membrane arm, including the matrix-facing CA module (orange) and the P2-domain (green), are highlighted. The suggested CA module is an assembly with triplets containing either CA1-CA1-CAL1 (or CAL2) or CA2-CA2-CAL1 (or CAL2) subunits.

Another unique feature of plant CI, compared with those in animals and fungi, is the presence of many additional subunits (Meyer, 2012; Braun *et al.*, 2014). These include several γ-carbonic anhydrase (CA)-related proteins, which form a matrix-exposed ‘egg-shaped’ module in the membrane arm (Fig. 1) (Sunderhaus *et al.*, 2006; Braun and Zabaleta, 2007). However, the function of this CI-related module remains unclear, and this is where the data presented by Córdoba *et al.* (2016) are novel and important.

## Mitochondrial biogenesis in germinating seeds

Respiration is key for successful germination and seedling development (Box 1). There are currently two models of mitochondrial biogenesis in germinating seeds: the ‘growth and division’ model and the ‘maturation’ hypothesis (Law *et al.*, 2014). According to the first model, the development of mitochondria occurs from pre-existing mature and fully functional organelles, already present in the dry seed. However, the latter theory proposes that mitochondria are disassembled during seed maturation, and then reassembled from the immature ‘pro-mitochondrial’ organelles found in the embryonic cells upon imbibition (Logan *et al.*, 2001).

**Box 1. Germination and respiration**

Mitochondria house the respiratory machinery and other metabolic processes essential for plant growth and development. Increased demands for metabolic energy are observed at different stages in the plant life cycle, but seem especially pronounced during the reproduction phase and germination. Upon fertilization, the seeds undergo a gradual maturation stage, in which the embryos develop and storage compounds (i.e. proteins, sugars or fatty acids) are synthesized in the maturing seeds. At maturity, the seeds are typically dispersed and enter the arrested mode (i.e. seed dormancy) where the embryo enters a ‘quiescent state’ (Bewley, 1997). Under these conditions seed respiration remains very low, most likely to preserve the stored energy reserves.

The timing of germination is critical for successful seedling development, and different environmental signals (e.g. water, temperature and light) are involved in breaking dormancy to enable germination. To achieve the high-energy demand during this critical stage, cellular respiration greatly increases shortly after imbibition, as the embryos develop. These processes may be accompanied by early biogenesis of the respiratory machinery.

This situation may resemble the differentiation of chloroplasts from non-photosynthesizing proplastids present in the embryos of mature seeds. In Arabidopsis, microarray data imply that mitochondrial biogenesis during embryo development involves the expression of numerous nuclear genes required in mitochondrial import and organellar gene expression, as well as many subunits of the respiratory machinery that are assembled with their related organelle-encoded counterparts upon their import into the organelle (Law *et al.*, 2014). The paper by Córdoba *et al.* (2016) offers additional insights regarding the biogenesis of CI during early embryogenesis in plants.

The CAs are zinc-binding proteins that catalyse the reversible interconversion of CO_2_ and water into HCO_3_^–^ and H^+^ (Braun and Zabaleta, 2007; Braun *et al.*, 2014). They fall into several distinct families (i.e. α, β, γ, δ and ε), which share only minor similarities in their sequences (possibly evolved independently though convergent evolution), and are mostly known with regard to CO_2_-concentrating mechanisms in the chloroplasts. However, their functions in other subcellular compartments, including mitochondria, are far less clear. Five different γ-CA subunits have been associated with the matrix-exposed CA-module in Arabidopsis mitochondria (Sunderhaus *et al.*, 2006; Braun and Zabaleta, 2007) (see also Fig. 1). CA1, CA2 and CA3 share a conserved active site, while the two remaining subunits are less similar and were therefore denoted as CA-Like proteins 1 and 2 (CAL1 and CAL2) (Braun and Zabaleta, 2007).

The physiological roles of the γ-CA subunits of plant CI are under investigation. Gene expression data indicate that both *ca1* and *ca2* are downregulated under high CO_2_, supporting a role in mitochondrial carbon metabolism. It was also noted that the mitochondrial γ-CA subunits may play important roles in photorespiration, mainly for the release of CO_2_ under high-light conditions (Braun *et al.*, 2014; Soto *et al.*, 2015). Genetic studies of various *ca* mutants indicate that the expression of mitochondrial γ-CAs is a prerequisite for CI biogenesis, seemingly at the earliest stages of the assembly of the enzyme (Braun *et al.*, 2014). Accordingly, CI exists only in residual amounts in Arabidopsis *ca2* and *ca3* mutants. Surprisingly, the phenotypes of the *ca* mutants are comparable with those of wild-type plants grown under similar conditions. Córdoba *et al.* (2016) indicate that while the functions of CA1 and CA2 are not essential during different stages in plant development, the double *ca1ca2* knockout affects embryo development, indicating redundancy between the two CA subunits in the mitochondria. The delayed embryo phenotypes of the double *ca1ca2* mutant are mainly associated with impaired mitochondrial membrane potential and increased levels of reactive oxygen species.

## The importance of respiratory complex I

How essential is plant CI to embryo development? It is a matter of controversy. The phenotypes of individual *ca* (and a few other) mutants affected in CI biogenesis are barely distinguishable from those of wild-type plants. However, CI deficiencies in many other plants strongly affect cellular physiology and plant development (although the plants are typically able to germinate and develop under normal growth conditions; see, for example, Keren *et al.*, 2012, and Cohen *et al.*, 2014). Arabidopsis mutants that are completely lacking CI activity, due to inactivation of the catalytic core subunit, NDUFV1, demonstrated severe growth and development defective phenotypes, but could otherwise be maintained in a viable state under specific growth conditions on sugar-containing synthetic media (Kuhn *et al.*, 2015). These data strongly suggest that CI functions are not essential in plants, probably due to the presence of alternative NAD(P)H-dehydrogenases that can bypass CI (Rasmusson *et al.*, 2008).

What is triggering the delayed embryo development phenotypes of the *ca1ca2* mutant? According to the model described by Córdoba et al. (2016), plant mitochondria may harbour different CI enzymes, according to the composition of their CA domains (i.e. the majority of CI, about 80%, is dependent on the presence of the CA2 subunit, while the remaining 20% are CA1-dependent forms). It was therefore suggested that no holo-CI could be formed in the absence of both CA1 and CA2 subunits. Interestingly, these may also coincide with the accumulation of various subcomplex I particles, observed in different plant CI mutants (Meyer *et al.*, 2011; Cohen *et al.*, 2014). Such CI assembly-intermediates may function incorrectly or interfere with normal respiratory activities. Alternatively, it remains possible that the phenotypes associated with the *ca1ca2* double mutant are unrelated to those of CI enzyme. Reduced (or no) CA activity in the *ca1ca2* mutant may affect the TCA cycle in the embryos due to altered CO_2_/HCO_3_^–^ ratios. However, any speculation regarding the roles of the CAs in mitochondrial carbon-metabolism needs to be supported experimentally.
